# Comparison of Two Synthesis Methods for 3D PLA-Ibuprofen Nanofibrillar Scaffolds

**DOI:** 10.3390/pharmaceutics17010106

**Published:** 2025-01-14

**Authors:** Esteban Mena-Porras, Annaby Contreras-Aleman, María Francinie Guevara-Hidalgo, Esteban Avendaño Soto, Diego Batista Menezes, Marco Antonio Alvarez-Perez, Daniel Chavarría-Bolaños

**Affiliations:** 1 School of Dentistry, Universidad de Costa Rica, Ciudad Universitaria Rodrigo Facio, San Jose 11501-2060, Costa Rica; mena2206@gmail.com (E.M.-P.); ancoby97@gmail.com (A.C.-A.); mfranciniegh@gmail.com (M.F.G.-H.); 2 Centro de Ingeniería y Ciencia de Materiales (CICIMA), Universidad de Costa Rica, Ciudad Universitaria Rodrigo Facio, San Jose 11501-2060, Costa Rica; esteban.avendanosoto@ucr.ac.cr; 3 Laboratorio Nacional de Nanotecnología (LANOTEC), Centro Nacional de Alta Tecnología (CENAT), San Jose 10109, Costa Rica; diegobatistadbm@gmail.com; 4 Tissue Bioengineering Laboratory, DEPeI-FO, Universidad Nacional Autónoma de México, Ciudad de México 04510, Mexico; 5 Programa de Posgrado en Odontología, Universidad de Costa Rica, Ciudad Universitaria Rodrigo Facio, San Jose 11501-2060, Costa Rica

**Keywords:** drug delivery system, ibuprofen, polylactic acid, scaffold, controlled release

## Abstract

Objectives: This study aimed to synthesize polylactic acid (PLA) nanofibrillar scaffolds loaded with ibuprofen (IBU) using electrospinning (ES) and air-jet spinning (AJS). The scaffolds were evaluated for their physicochemical properties, drug release profiles, and biocompatibility to assess their potential for local analgesic applications. Methods: Solutions of 10% (*w*/*v*) PLA combined with IBU at concentrations of 10%, 20%, and 30% were processed into nanofibrillar membranes using ES and AJS. The scaffolds were characterized using scanning electron microscopy (SEM), differential scanning calorimetry (DSC), thermogravimetric analysis (TGA), and Fourier-transformed infrared (FT-IR) spectroscopy. The drug release profile was assessed by ultraviolet-visible spectrophotometry (UV-Vis), and cell adhesion and viability were evaluated using fibroblast culture assays. Statistical analyses included qualitative analyses, *t*-tests, and Likelihood ratio tests. Results: SEM revealed randomly arranged nanofibers forming reticulated meshes, with more uniform dimensions observed in the AJS group. TGA and DSC analyses confirmed the thermodynamic stability of the scaffolds and enthalpy changes consistent with IBU incorporation, which FT-IR and UV-Vis validated. Drug release was sustained over 384 h, showing no significant differences between ES and AJS scaffolds (*p* > 0.05). Cytotoxicity and cell viability assays confirmed scaffold biocompatibility, with cellular responses proportional to drug concentration but within safe limits. Conclusions: PLA-IBU nanofibrillar scaffolds were successfully synthesized using ES and AJS. Both methods yielded biocompatible systems with stable properties and controlled drug release. Further, in vivo studies are necessary to confirm their clinical potential.

## 1. Introduction

The design of scaffolds for tissue engineering with analgesic capacity is an innovative concept with significant potential in oral health [1]. These designs offer the interaction between progenitor cells, regulatory signals, and the biomaterials/scaffolds used to deliver them (known as the tissue engineering triad), adding an analgesic benefit [2]. Studies suggest the design of three-dimensional structures, commonly referred to as “membranes”, capable of fulfilling biological, mechanical, and morphological requirements [3] to create biomechanical support, which allows for local drug administration in the oral cavity and promotes tissue regeneration [4,5]. Scaffolds can be functionalized according to clinical needs, sometimes with growth factors, bioactive molecules that induce mineralization, or drugs with antibiotic or analgesic potential [6]. Other functions include biomechanical support, facilitating cell adhesion, allowing diffusion of nutrients or waste, ensuring biocompatibility, and enabling bioresorption [3,4,6,7,8]. More recently, scaffolds have incorporated molecules like graphene to treat nerve injuries [9] or cancer treatment [10].

Natural polymers, such as collagen, chitosan, cellulose, silk fibroin, and alginate, are commonly used as scaffold manufacturing materials [11,12]. Synthetic polymers include polycaprolactone, polyglycolic acid, polyethylene glycol, lactic acid, and polyurethane [5,13]. Polylactic acid (PLA), a linear aliphatic polyester derived from renewable resources, is cost-effective and characterized by properties like absorbability and non-toxicity after degradation, making it highly sought after for scaffold production [14,15,16]. Additionally, PLA has been widely approved by the United States Food and Drug Administration (FDA), making it suitable and safe for all applications involving direct contact with biological fluids [17].

Several methods can be used to create bone tissue scaffolds with tridimensional structures, such as the foam replica method, electrospinning, air jet spinning, freeze-drying, gas foaming, solvent casting/particulate leaching, phase separation, and molecular self-assembly [15,18,19,20]. These include the foam replica method, electrospinning, air jet spinning, freeze-drying, gas foaming, solvent casting/particulate leaching, phase separation, and molecular self-assembly [15]. Due to its rapid prototyping capabilities, 3D printing technology revolutionizes scaffold development, though this method requires expensive equipment [16,21]. Air-Jet Spinning (AJS) and Electrospinning (ES) techniques produce submicron or nanoscale fibers from synthetic and natural polymers. The fiber morphology can be controlled by various parameters such as polymer concentration, surface tension, working distance, spinning gas, temperature, gas pressure, and evaporation rate [22,23]. AJS technology offers several advantages, such as a faster, easier to use, less expensive, and safer method, due to the absence of high voltage, scalability, and versatility in solvent choice. It can also be used to produce micro- or nanoscale fibers from various polymers [24]. The ES technique offers additional benefits, such as simplicity, adaptability, and versatility [25,26]. A wide range of polymers can be electrospun, and the resulting fibrous structures, known as “scaffolds”, have been shown to effectively mimic the extracellular matrix during the culture process of various cell types [27].

Emerging trends in pharmacology are shifting from systemic to local drug administration, which opens the possibility of loading these scaffolds with different drugs to provide localized functionality [28,29]. Studies have explored combinations of various synthetic materials, such as polycaprolactone, PLA, and PLGA, with substances that enhance bone formation, such as hydroxyapatite, and drugs that provide analgesia [5,12,13,28,29,30]. This is particularly relevant for drugs with significant adverse effects when administered systemically, such as non-steroidal anti-inflammatory drugs (NSAIDs) [31,32,33].

NSAIDs are part of a heterogeneous group of drugs that have analgesic, antipyretic, and anti-inflammatory properties, falling between corticosteroids (with anti-inflammatory properties) and opioids (considered major analgesics) [34]. Traditional NSAIDs inhibit cyclooxygenase isoforms (COX-1 and COX-2), enzymes mediating arachidonic acid’s conversion to prostaglandins and prostacyclin [35,36]. COX-1 is constitutive and responsible for producing prostaglandins involved in homeostatic processes such as gastrointestinal cytoprotection, while COX-2 produces prostaglandins associated with pain and inflammation. The physiology and adverse effects of NSAIDs are linked to their selectivity for COX-1 or COX-2 [36,37]. Ibuprofen (IBU) (2-[4-(2-methylpropyl) phenylpropanoic acid]), an NSAID derived from propionic acid, is characterized by moderate efficacy. It acts through non-specific, reversible inhibition of COX-1 and COX-2, providing analgesia and reducing edema and the immune response to inflammation [38,39]. With a molecular weight of 206.28 g/mol, IBU is considered slightly soluble in water due to the presence of nonpolar alkyl groups and the benzene ring, which reduce its polarity, and due to its solubility (0.011 mg/mL at 25 °C and pH 7.4), it is considered more soluble in alcohols [40]. IBU contains two main functional groups: the carboxylic acid group (COOH) and the aromatic ring (benzene) [41,42]. Studies on the melt-crystallization of IBU confirm that its glass transition temperature is −42.3 °C, with a melting point of 78.9 °C [43,44].

Local formulations of NSAIDs produce a site-specific effect while minimizing systemic undesirable side effects in patients, such as gastrointestinal affection, nephrotoxicity, or cardiotoxicity [28,29,31,45,46]. The local application of IBU for musculoskeletal pain is comparable to other topical agents, such as diclofenac and ketoprofen. Local IBU formulations include supersaturations, microemulsions, nanosystems, gels, and microneedles, with most containing 5% by weight of IBU [45,47]. Although previous efforts have been made to create IBU-PLA mats fabricated by ES, there are no previous reports employing AJS, nor have there been any comparisons between both techniques [48]. Thus, this study aimed to synthesize PLA fibrillar spun mats loaded with IBU by ES and AJS techniques and to analyze its physicochemical, release profile, and cell biocompatibility properties.

## 2. Materials and Methods

### 2.1. Fabrication

Poly(lactic acid) pellets (C_3_H_6_O_3_; molecular weight of 192,000 (called Ingeo 2003D were purchased from Promaplast, Mexico City, Mexico) were dissolved 24 h in chloroform and ethanol in a 3:1 ratio to create a 10% (*w*/*v*) PLA solution (control). Subsequently, (S)-(+) IBU (Sigma-Aldrich, St. Louis, MO, USA) was added to the 10% PLA control solution at concentrations of 10%, 20%, and 30 wt% relative to polymer weight. The mixture was done at 300 rpm for 4 h. A pilot test was performed to assess whether the order of PLA and IBU mixing affected the drug’s incorporation into the membrane. During the preparation of the solutions and synthesis processes, residual materials at the bottom of the beakers were collected and referred to as “residuals” of the membranes. The methodology for both techniques was selected and adjusted, according to previous reports [3,16,49,50].

For the AJS technique, the solutions were loaded into a commercially available airbrush (Toolcraft, model TC4176) with a 0.3 mm diameter nozzle and a 7 mL reservoir pre-purged with acetone. The airbrush was connected to a compressed air line with a firing pressure of 30 Psi. A 35 cm × 35 cm aluminum collector plate was placed 10 cm from the airbrush’s firing tip. The solution was sprayed manually, moving the airbrush back and forth horizontally for 10 min.

For the ES technique, a needle with an internal diameter of 0.508 mm was attached to the end of a syringe containing the ibuprofen/PLA solution. A total of 7.75 mL (±0.05 mL) of the solution was electrospun at a constant feed rate of 1 mL/h for 8 h. Electrospinning was conducted horizontally, using a high-voltage source connected to a grounded collector plate. A 15 kV charge was applied, drawing the polymer solution from the syringe to the collector plate. The nanofibers were produced at room temperature 24 °C (±2 °C) with approximately 50% (±5%) humidity. Once obtained, the scaffolds were characterized according to previous investigations [3,16].

### 2.2. Morphological Characterization

The microstructure morphology of the scaffolds was analyzed using scanning electron microscopy (SEM) (JEOL JSM-6390LV, Tokyo, Japan) with a 10 kV acceleration voltage. The samples were sputter-coated with a 20 nm thin layer of gold for 180 s using a Denton Vacuum Desk V. This allowed for analysis of both the outer and inner surfaces. Image J software Version 1.54g was used to determine membrane thickness, fiber dimensions, and pore sizes.

### 2.3. Physicochemical Characterization

#### 2.3.1. Differential Scanning Calorimetry (DSC)

Three samples of pure PLA and IBU and the obtained scaffolds (each weighting 2.0 mg (±0.2 mg)) were placed in sealed aluminum DSC crucibles. The samples were analyzed using a DSC unit Q200 (TA Instruments, New Castle, DE, USA). Thermal scans were initiated at 25 °C, and heating continued at a rate of 10 °C/min until a final temperature of 200 °C was reached. Data were analyzed using Universal Analysis 2000 software (version 4.5A, TA Instruments) to determine the glass transition temperature (Tg) and melting point (Tm). Results were obtained from the first heating cycle.

#### 2.3.2. Thermogravimetric Analysis (TGA) and Differential Thermogravimetric Analysis (DTG)

Three samples of pure PLA and IBU were placed in TGA pans, and the obtained scaffold samples weighting 2.0 mg (±0.2 mg). The analysis was performed starting at a base temperature of 25 °C for three minutes and heated in a constant ramp from from 25 °C to 700 °C at a rate of 20 °C/min. On set point (To), inflection point (Tp), and maximum weight loss (Tmax) were determined. Data were analyzed using Universal Analysis 2000 software (version 4.5A, TA Instruments).

#### 2.3.3. Transform Infrared Spectroscopy (FTIR)

Three samples of pure PLA and IBU, as well as the scaffolds that were obtained, were analyzed using an FT-IR Nicolet 6700 spectrometer to assess the infrared absorption spectra. The data were evaluated using OMNIC spectra 32 analysis software. Spectra were corrected and linearized to identify the main signals across the spectra. Comparative analyses were done to evaluate the presence of the IBU within the scaffolds and possible chemical changes in the polymeric matrix.

#### 2.3.4. Ultraviolet-Visible Spectroscopy (UV-VIS)

The calibration curve for IBU in phosphate-buffered saline (PBS, pH = 7.4) mixed with ethanol in a 5:1 ratio was calculated using UV-VIS analysis (Genesys 150 Thermoscience System). Then, a controlled release assay was performed employing Transwell cell culture plates (Corning Inc., Corning, NY, USA) to evaluate the release profile of the scaffolds in different periods. Aliquotes collected from the reservoir were collected and stored at 4 °C until the end of the experiment. Finally, 2 mL of fresh PBS-Ethanol 5:1 combination was combined with 1 mL from each aliquote to obtain a final 3 mL volume to be analyzed. The IBU absorbance was measured at a wavelength of 264 nm over 2, 4, 8, 24, 48, 72, 96, 168, 240, 312, and 384 h. Each scaffold was evaluated in triplicate.

### 2.4. Cell Proliferation Assay

The obtained scaffolds were sterilized by ultraviolet light for 30 min in 48-well plates. After sterilization, 1 × 10^5^ cells/mL of human fetal osteoblast (hFOB) cells were seeded onto the scaffolds and incubated for 30 min. Following this, 400 μL of DMEM medium supplemented with 10% FBS was added, and the culture plates were maintained at 37 °C in an incubator with a 5% CO_2_ atmosphere for 2, 4, and 6 days. Cell proliferation was evaluated using the WST-1 kit (Cell Proliferation Reagent WST-1, ROCHE, Basel, Switzerland) by adding 40 μL of the reagent (1:10 concentration). The cultures with WST-1 were incubated for 4 h, and absorbance was measured at 450 nm using a ChroMate 4300 AWARENESS plate reader. The culture dish served as a positive control.

### 2.5. Statistical Analysis

Qualitative analyses were performed for data obtained from DSC, TGA, and FT-ir analyses. All quantitative data were expressed as the average ± standard error of the mean. Numerical data were analyzed via Student’s *t*-test to determine the differences among the groups. The *t*-student test analyzed data of the fibers diameter and the “Likelihood ratio test” was used to compare fiber dimensions and the drug release profile statistically. The R Console program, version 4.1.2 (1 November 2021)—“Bird Hippie”, was employed for statistical analysis. Statistical significance was considered at *p* < 0.05.

## 3. Results

### 3.1. Morphological Characterization

**Figure 1:**
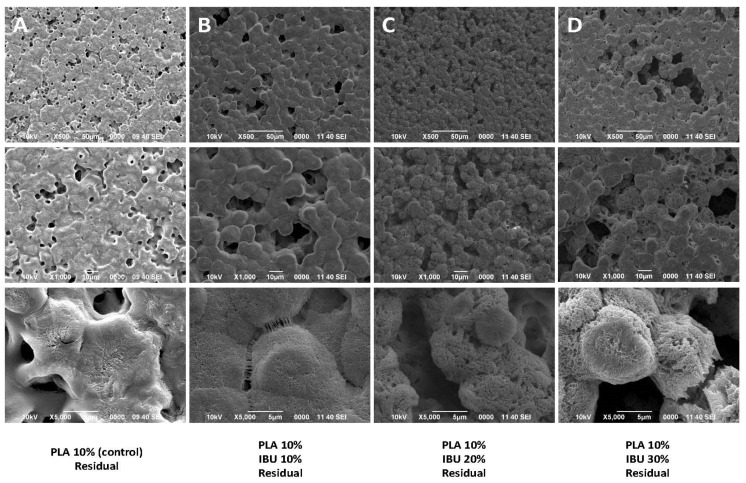
SEM images showing the morphology of the (**A**) PLA 10% control and PLA 10% with (**B**) 10%, (**C**) 20%, and (**D**) 30% Ibuprofen residuals. SEM images are shown at 500×, 1000×, and 5000×. (unclear word in (**A**) bottom is 5 µm).

SEM analysis of the residuals (Figure 1) reveals the formation of a film characterized by spherical structures rather than fibrillar ones. It can be observed that when PLA and IBU are dissolved together, the drug crystals are no longer visible, suggesting successful incorporation of the drug into the polymer matrix.

**Figure 2:**
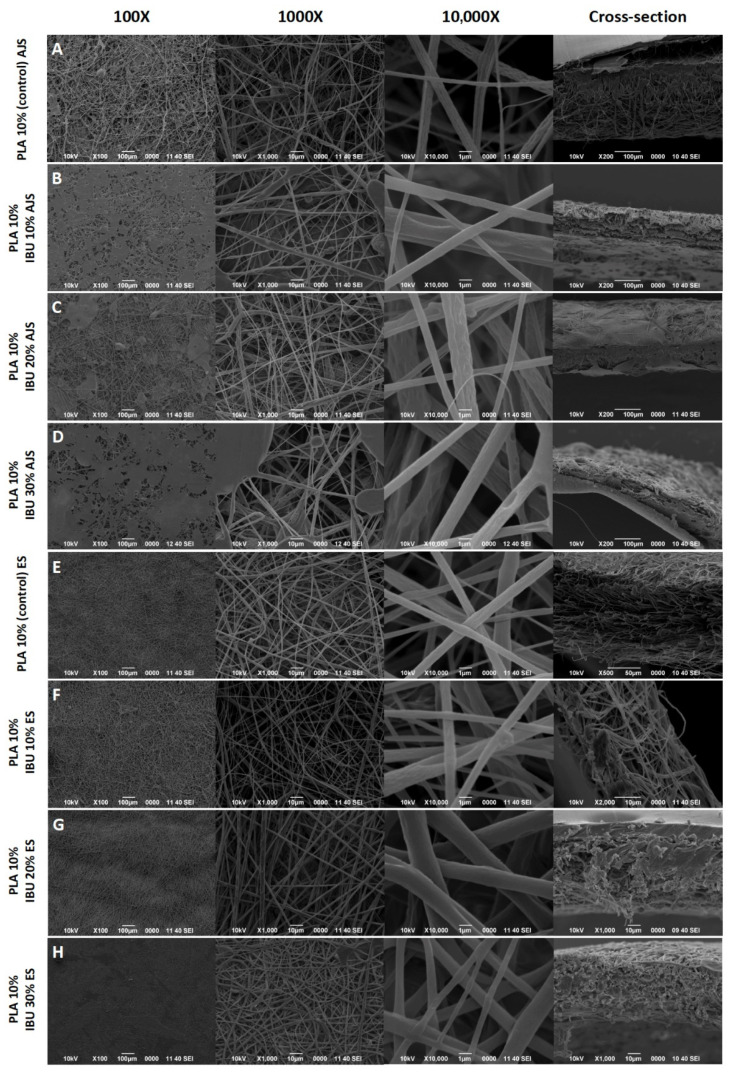
SEM images showing the morphology of PLA 10% scaffolds at 100×, 1000×, and 10,000× and their cross-section. Membranes of (**A**) 10% PLA (control) and 10% PLA with (**B**) 10, (**C**) 20, and (**D**) 30% Ibuprofen synthesized with the AJS technique are observed. Also, membranes of (**E**) PLA 10% (control) and PLA 10% with (**F**) 10, (**G**) 20, and (**H**) 30% Ibuprofen synthesized with the ES technique are shown.

Figure 2 shows the membranes synthesized by AJS and ES. SEM analysis revealed the presence of randomly arranged micro- and nanofibers forming a reticulated mesh with no gaps or bare areas. The average sizes of the AJS fibers were 0.9804 µm (±0.33 µm) and 1.11805 µm (±0.30 µm) for the ES fibers. However, larger or smaller fibers were present in some areas, along with the formation of honeycomb-like structures. The pore size of the fibers was consistent across all samples. Cross-sectional analysis revealed that fibers synthesized by AJS technique are thicker membranes than those obtained by ES, with average thicknesses of 192,24 (±103.01) µm and 70 (±37.87) µm, respectively.

**Figure 3:**
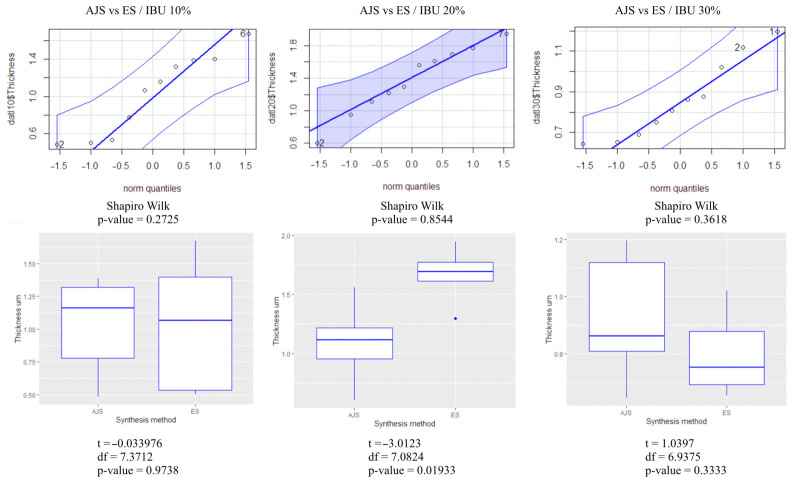
Statistical analysis of fiber thickness in 10% PLA membranes with 10, 20, and 30% IBU.

Fiber thickness between the two spinning methods was analyzed using the *t*-test and box plot analysis (Figure 3), with quantile comparisons (ggplot graphs). The normality of the results was analyzed by the Shapiro-Wilk test. It was determined that the 10% and 30% fiber groups showed no significant difference between the two methods (*p* > 0.05). However, the 20% fibers rejected the null hypothesis (H0) (*p* < 0.05), indicating a statistically significant difference between the AJS and ES synthesis methods.

### 3.2. Physicochemical Characterization

#### 3.2.1. Differential Scanning Calorimetry (DSC)

**Figure 4:**
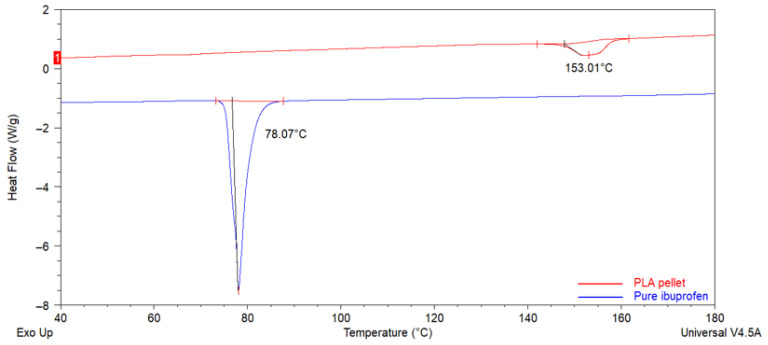
DSC thermograms of the PLA pellet and pure Ibuprofen’s control settings for thermal characterization.

**Figure 5:**
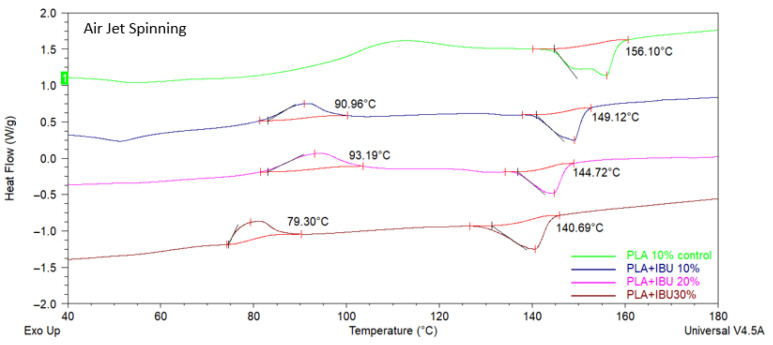
DSC thermograms of the PLA 10% control scaffold and PLA + IBU 10, 20, and 30% scaffolds synthesized by Air Jet Spinning.

**Figure 6:**
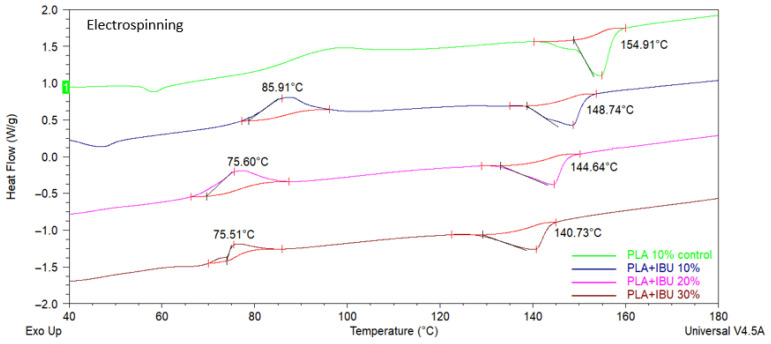
DSC thermograms of the PLA 10% control scaffold and PLA + IBU 10, 20, and 30% scaffolds synthesized by Electrospinning.

Figure 4 shows DSC curves for both groups. Endothermic signals are identifiable for pure PLA and IBU, indicating melting temperature (Tm) of 78 °C and 155 °C for IBU and PLA, respectively. Figure 5 and Figure 6 show the analyses for 10% PLA membranes and PLA membranes charged with IBU at three concentrations (10%, 20%, and 30%). Charged membranes showed thermal changes close to the IBU Tm, suggesting the presence of the drug. A progressive decrease in Tm and Tg for pure PLA membranes values was observed. Shifting of the PLA Tm in function of IBU concentration also suggests the homogeneous incorporation of IBU onto the membranes. No impurities were observed in the analyzed samples. No observable differences were detected between the DSC profiles for AJS and ES membranes.

**Figure 7:**
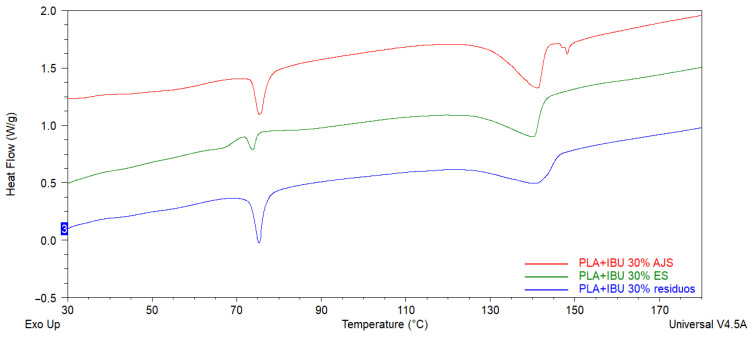
DSC of residual and pilot test membranes synthesized with AJS and ES, loaded with 30% ibuprofen.

Additionally, DSC thermograms for residuals were evaluated (Figure 7). The results indicate that if IBU and PLA combine only mechanically, the drug maintains its physical form, with apparent visible Tm. This result confirms that both AJS and ES synthesis techniques improved the incorporation of the drug into the polymeric matrix.

#### 3.2.2. Thermogravimetric Analysis (TGA)

**Figure 8:**
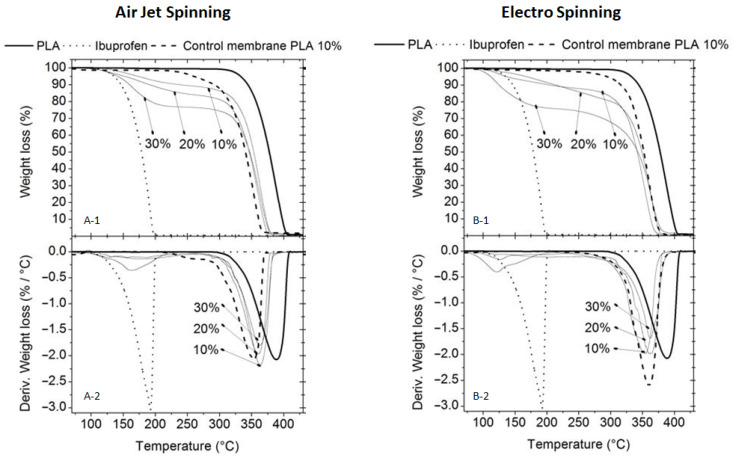
Thermogravimetric analysis (TGA) thermograms of PLA Pellet and Pure Ibuprofen vs. AJS PLA10% + IBU membranes ((**A-1**) % weight loss and (**A-2**) DTG curves). PLA Pellet and Pure Ibuprofen vs. ES PLA10% + IBU membranes ((**B-1**) % weight loss and (**B-2**) DTG curves).

TGA and DTG analyses revealed that both synthesis techniques (AJS and ES) exhibited similar thermal degradation patterns (Figure 8). The PLA membranes showed Tmax around 400 °C, whereas the degradation of IBU was evident near 200 °C. A decrease in both the To and Tmax was observed for the charged membranes, with all membranes degrading below 400 °C. This indicates the incorporation of IBU and demonstrates that the drug reduces the Tmax of pure PLA. DTG analyses further confirmed this observation, as the Tp for the charged membranes occurred near and below the Tp of pure PLA. Notably, these changes depended on the IBU concentration; higher IBU concentrations resulted in more pronounced shifts in the thermal signals towards those characteristic of pure IBU.

#### 3.2.3. Transform Infrared Spectroscopy (FTIR)

**Figure 9:**
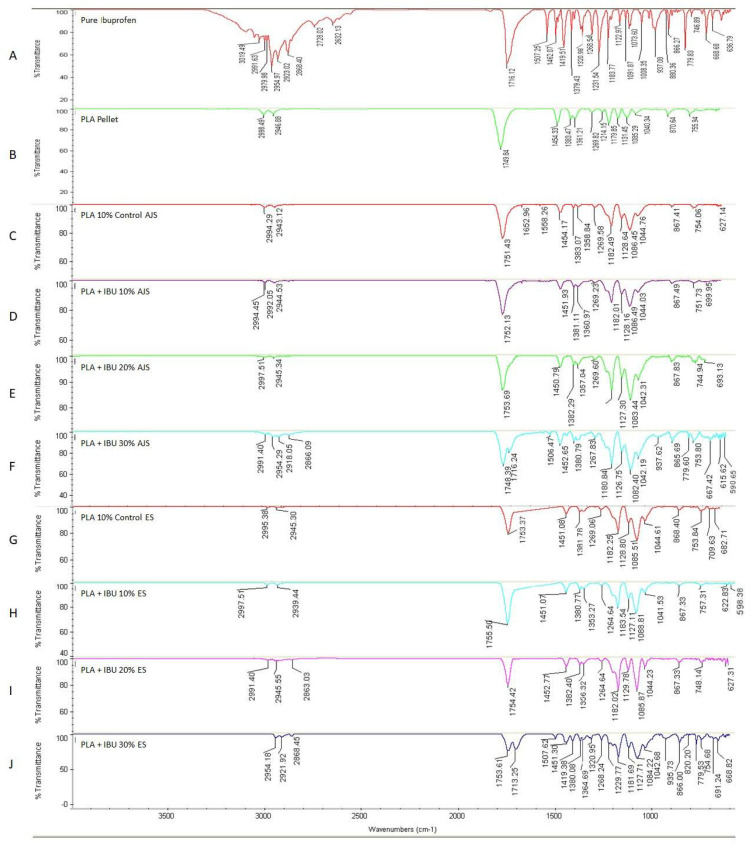
FT-IR Spectroscopy of (**A**) Pure ibuprofen, (**B**) PLA pellet, (**C**) PLA10% control, (**D**) PLA + IBU 10%, (**E**) 20%, (**F**) 30% scaffolds done by Air jet Spinning and (**G**) PLA10% control, (**H**) PLA + IBU 10%, (**I**) 20%, (**J**) 30% scaffolds done by Electrospinning.

Regarding the chemical profile, in both techniques, the incorporation of IBU does not alter the infrared profile of the PLA (Figure 9). When analyzing the entire spectrum, new absorbance peaks are detected in all IBU-loaded scaffolds compared to the PLA control membranes for both spinning methods. These peaks are associated with the functional groups observed in IBU spectra. The following strong signals from IBU were identified in the infrared spectrum: i. Asymmetric stretching of the methyl group (CH_3_) at 2955 cm^−1^, ii. Stretching of the carbonyl group (C=O) at 1721 cm^−1^ and 1231 cm^−1^, and iii. Oscillating vibrations of the methylene group (CH_2_) at 779 cm^−1^.

Additionally, medium-strength signals were observed at 1268, 1380, 1506, and 2869 cm^−1^, corresponding to methyl, alkene, and hydroxyl groups. These signals are primarily observed in scaffolds loaded with 30% IBU; in other membranes, the signals are less detectable due to the lower proportion of the drug. However, the signals indicate the successful incorporation of IBU into the membrane.

#### 3.2.4. Ultraviolet-Visible Spectroscopy (UV-VIS)

**Figure 10:**
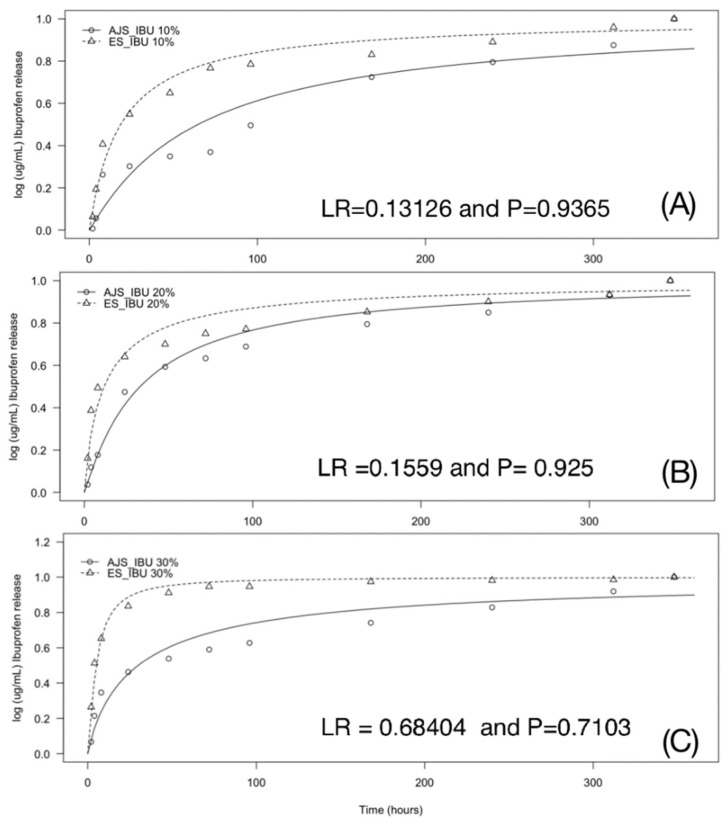
Comparison of 10%, 20%, and 30% of Ibuprofen release concentrations in PLA membranes according to the Air Jet Spinning and ElectroSpinning technique. (**A**) IBU 10%, (**B**) IBU 20%, (**C**) IBU 30%.

The controlled release assay conducted over 2, 4, 8, 24, 48, 72, 96, 168, 240, 312, and 384 h showed that IBU-PLA membranes obtained by both synthesis methods can release the drug over time (Figure 10). A comparison of the IBU release profiles between the membranes manufactured using the AJS and ES techniques was performed using the Likelihood Ratio test (LR). The results indicated that when modeling IBU release at the same concentrations, all LR values were (*p* > 0.05), suggesting no statistically significant difference between the two groups (AJS and ES) for the concentrations evaluated. Furthermore, the graph illustrates that as the concentration of IBU increases, a more significant percentage of the drug is released in less time.

### 3.3. In Vitro Studies

**Figure 11:**
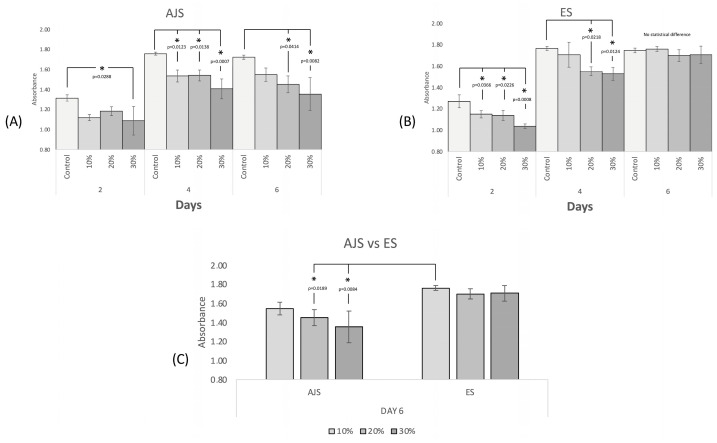
Proliferation of osteoblasts at 2, 4, and 6 days to the exposure of (**A**) AJS and (**B**) ES membranes. (**C**) Comparison of cell proliferation after 6 days of AJS vs. ES. (* Significant statistical difference).

Cell proliferation tests using hFOBs (Figure 11) demonstrated that all IBU-loaded membranes were biocompatible, supporting continued cell growth. By day 6 of the test, an increased number of cells was observed across all concentrations and fabrication techniques evaluated compared to day 0. The 10% group exhibited an enhanced biocompatible response, displaying behavior similar to that of the control group in both methods by day 6. However, while the ES technique showed no significant differences between IBU concentrations, the AJS technique revealed a decreased cell response as the IBU concentration increased. These findings suggest that ES membranes offer more predictable behavior. In contrast, with the AJS technique, differences were observed as early as day 2 between the control group and the 30% group. By day 4, all AJS groups were statistically different compared to the control.

## 4. Discussion

Developing a drug delivery system that enables the local release of an NSAID while promoting cell proliferation is a promising strategy for treating conditions where tissue regeneration is accompanied by pain and inflammation. This study focused on synthesizing PLA fibrillar spun mats loaded with IBU using AJS and ES. Additionally, it evaluates the potential advantages and limitations of these methods concerning the physicochemical characteristics, release profiles, and biocompatibility of the resulting products. Any material intended for general or professional use must be validated before commercialization [51]. In our in vitro assays, we aim to validate whether the manufacturing process of these specific scaffolds influences their properties. Designing a study to compare both synthesis methods is particularly important from a cost-feasibility perspective in production. While ES offers more predictable results due to the absence of human error, it requires more expensive equipment and is more time-consuming. Conversely, AJS may provide a faster, more accessible, and cost-effective alternative; however, its reliance on manual production introduces variability that could affect the expected results.

As an initial step, confirming the effective incorporation of IBU into the polymer matrix was necessary to ensure the production of nanofibrillar spun mats without alterations. Nanofibrillar materials offer several biological advantages, including structural support, facilitation of cell adhesion, functionalization potential tailored to clinical needs, efficient diffusion of nutrients and waste, and compatibility with surrounding tissues [3,6]. In this case, these features are coupled with a controlled release environment for IBU.

After mechanically combining PLA and IBU using controlled solvents and magnetic stirring, no fibers, crystalline structures, or pores were observed in the residuals under SEM imaging at various magnifications. The DSC analysis revealed a thermal profile showing the Tm of pure IBU at approximately 78 °C, consistent with the findings reported by Lee et al. [43]. According to Belmessaoud et al., when a drug is incorporated into a polymer matrix, the Tm signal of the drug may be attenuated due to its dispersion within the polymer [52]. Our residuals further confirmed this, where pure IBU signals remained detectable but decreased. However, after the membrane synthesis, the integration of IBU into the polymer matrix was improved. This successful incorporation can be attributed to the amorphous state assumed by IBU during the dissolution phase and the development of specific interactions between PLA and IBU, which inhibit crystal growth [43,52,53]. Therefore, the sequence of compound incorporation during the dilution process and the synthesis method is critical to ensuring the proper integration of IBU into the matrix.

Morphological characterization of membranes, including porosity and structural nanofibers, is a critical consideration in the design and synthesis of biomaterials [54]. The porosity of scaffolds directly influences their functionality in biomedical applications. Open, porous, and interconnected networks are essential for supporting cell nutrition, proliferation, and migration—key processes required for tissue vascularization and the formation of new tissues. Moreover, the network structure of pores plays a pivotal role in guiding and promoting new tissue formation [55]. The morphology and diameter of the fibers obtained in this study align with those reported by Solarz et al. and Granados et al. Under similar conditions, incorporating a new component into the fibers resulted in more cylindrical shapes with no surface pores and an increase in average fiber diameter [56,57]. Similarly, M. Mohiti-Asli et al. observed that adding IBU to a polymeric system reduced the presence of pores as they were filled with the drug, depending on its concentration [30].

Regarding fiber diameters, significant differences between methods were observed at 20% concentration. However, the current literature does not explain this variation. It is possible that the analyzed fibers were more heterogeneous, but this variability is unlikely to have impacted the synthesis process, as all fibers were produced under the same parameters. Furthermore, according to Solarz et al., the morphology and diameter of synthesized fibers can be affected by factors such as the distance between the needle tip and the collector during electrospinning. Additionally, using the ImageJ program to measure pore size from 2D SEM images may not fully capture the three-dimensional pore distribution throughout the sample depth [56].

Thermodynamic characterization suggests successfully loading the drug into the polymeric matrix [58]. DSC revealed changes in the enthalpy of the membranes, with the absence of the endothermic peaks associated with the Tm of pure IBU [59,60,61]. Additionally, an increase in the exothermic peak was observed. According to Gómez et al., this behavior is attributed to higher temperatures enhancing the mobility of polymer chains, allowing their reorganization into crystalline structures—a common phenomenon in polymers such as PLA [61]. Moreover, the crystalline nature of IBU enables it to form additional crystalline structures upon heating, resulting in the exothermic peak associated with crystallization (Tc) [44,62]. The interaction between PLA and IBU, each with distinct structures, facilitates the incorporation of both components. This interaction contributes to the controlled stabilization of IBU, as the polymer matrix shields it, preserving its integrity. This distinction in crystallization is evident in the synthesized systems [61]. A change in the thermal capacity of the scaffolds was also observed. As the IBU concentration increased in the experimental groups, the melting and crystallization signals shifted to lower temperatures, indicating the drug’s and the polymer’s interaction. This shift in enthalpy is likely linked to the hygroscopic properties imparted by the increased drug concentration, which affects the membrane’s moisture absorption capacity and alters its melting point [63]. A similar phenomenon has been reported for ketoprofen and dexketoprofen trometamol, where progressive reductions in enthalpy changes were associated with higher drug concentrations. Increased drug content tends to enhance the mechanical and thermal properties of the system [64].

TGA confirmed that the membranes loaded with IBU maintain thermal stability, with values similar to those of the control membrane and pure PLA, aside from changes attributed to the presence of IBU. The analysis revealed that PLA undergoes a single degradation step, with maximum weight loss occurring at approximately 373 °C. This corresponds to the breakdown of polymer bonds, releasing gaseous products such as cyclic oligomers, acetaldehyde, lactide, and carbon monoxide [62]. In contrast, the thermal degradation of pure IBU begins at 114 °C and reaches its maximum weight loss at 200 °C, consistent with the findings of Belmessaoud et al. [52]. Two distinct degradation steps are observed when IBU is incorporated into the membranes: the first associated with IBU and the second with PLA. Additionally, a continuous decrease in the mass loss temperature is observed with increasing IBU concentration [52,65]. DTG further illustrates these observations, particularly when analyzing Tp’s. It was evident that the Tp of the loaded membranes shifted to lower values (left) as a function of IBU concentration. In the 30% group, a more pronounced second Tp corresponding to IBU was observed. These changes were consistent across both synthesis methods. Interestingly, the degradation temperature of IBU molecules in the loaded membranes shifts to higher values than pure IBU, suggesting the successful incorporation of IBU into the pores of the PLA fibers. According to Namazi et al., this phenomenon arises from the restricted molecular movement of IBU molecules within the pores and hydrogen bonding interactions between hydroxyl (OH) groups in the pore walls and carboxyl (COOH) groups of the IBU molecules. These interactions reduce the vapor pressure of the embedded IBU molecules, thereby increasing their degradation temperature [66].

Drug loading was confirmed by FT-IR analysis, indicating that the incorporation of IBU is compatible with the polymer system and does not alter the infrared profile of PLA. The characteristic peaks of IBU remain visible, suggesting successful integration. The observed transmittance values align with findings previously reported, as the spectra associated with IBU’s functional groups fall within the range of 4000–400 cm^−1^ [67,68,69]. Furthermore, according to Lemraski et al., the bands in the 2540–3098 cm^−1^ range, representing the stretching of the alkyl group—a characteristic marker of ibuprofen—were present in all synthesized samples [70].

UV-Vis spectroscopy confirmed the presence of IBU in aliquots collected over a 384-h period, with absorbance observed at 264 nm, similarly to previous reported investigations [32,41]. This finding indicates that the system successfully incorporates IBU into the membranes and demonstrates its capacity for controlled drug release over time. The IBU was recovered through hydrolysis of the system. Rodríguez highlights that ibuprofen hydrolysis can yield enantiomers with high purity [71]. In this study, for both groups (AJS and ES), up to 85% of the IBU was released within the first 100 h, with no statistically significant differences between the two techniques [72,73]. Regarding the release medium, Riggin et al. also used PBS in their in vitro tests and found that IBU release followed a linear profile in saline solutions. However, in vivo or in vitro serum tests showed a rapid release profile, indicating that the surrounding environment influences scaffold release properties [74]. For comparison, Lima et al. utilized latex membranes to control IBU, observing a stable release profile with 60% released in an alkaline medium and 50% in an acidic medium [75]. These release percentages are lower than those observed in this study, suggesting that testing in environments other than physiological ones can yield different drug recovery results. This is particularly relevant in different environments, such as the oral cavity, where basic and acidic conditions can occur depending on the degree of inflammation or infection.

In the cell proliferation test, the primary concern was the impact of IBU on PLA scaffolds, particularly given that the G0/G1 phase of osteoblasts is associated with bone density loss when NSAIDs are systemically consumed [76,77]. However, García et al. report that IBU does not impair the proliferative capacity of osteoblasts at therapeutic doses [78]. Few studies in the literature have assessed the effect of controlled local release of IBU on bone cells. Limami et al. developed calcium phosphate granules loaded with IBU as a bone substitute and local analgesic for osteoarthritis. Their in vivo study demonstrated that IBU-loaded and unloaded calcium phosphate granules promoted progressive osteogenesis [79]. Similar to our findings, this suggests that low doses of locally administered IBU are compatible with osteoblast proliferation. Our results indicated that the selected IBU concentrations did not induce cell toxicity. However, higher concentrations reduced the proliferation capacity of hFOBs during the first four days. All membranes exhibited similar behavior by day six regardless of the synthesis method. Notably, the 10% IBU concentration group showed the highest biocompatibility by day six, with results comparable to the control group for both techniques. The ES group demonstrated more stable performance than AJS, suggesting improved controlled clinical behavior during the initial four days. If a concentration greater than 10% is required, the synthesis method could have a significant clinical impact, making ES the preferred method. Conversely, if higher concentrations are unnecessary, no significant differences exist between the two methods, and AJS may be favored for its faster production and lower cost. Further, in vivo studies are required to evaluate the clinical performance of different concentrations and synthesis methods.

A drug delivery system for IBU offers significant advantages, particularly when enhanced local bioavailability is desired. According to the presented results, these customized scaffolds employing PLA can be obtained either by AJS or ES, with minimal differences between the final products. Plasma concentrations of orally administered IBU are approximately 300 times higher than locally delivered ones. Moreover, only 0.55% of locally administered IBU is excreted in the urine after 24 h, compared to 97% for orally administered ibuprofen [42]. Side effects from topical NSAIDs occur in 10–15% of patients, with gastrointestinal effects being rarely reported [34,42,80]. Although limited literature exists on the renal and cardiovascular side effects of local NSAIDs, toxic systemic levels are rarely reached after local administration [42,80]. The proposed PLA-IBU scaffold presents potential clinical benefits aligning with the peak period of acute postoperative pain. According to the Visual Analogue Scale (VAS), moderate, severe, and unbearable pain intensity is reported in 60–66.3% of patients postoperatively during the first 24 h [81,82]. In the context of dental pain, postoperative pain typically reaches its maximum intensity within the first 12 h after surgery, with most pain associated with oral surgeries subsiding within the first week [83,84]. Consequently, the synthesized scaffolds may provide significant analgesic and anti-inflammatory benefits during this critical period. This timeframe is further supported by Malamed’s study, which indicates that pain following dental procedures peaks on the first postoperative day, with the highest levels of analgesic consumption occurring within the first 48–72 h after third molar extractions [85]. Although these results offer promising clinical advantages, important limitations must be addressed in future investigations. First, the release experiment should be repeated under dynamic conditions that mimic tissue environments, such as exposure to blood, enzymatic activity, varying temperatures, and different pH levels. Second, validating whether the tested IBU concentrations can provide an improved local analgesic effect using specific in vivo pain models is necessary. Finally, the proper clinical application of the scaffolds synthesized by both methods will ultimately depend on comprehensive in vivo evaluations and human clinical trials.

## 5. Conclusions

This study successfully demonstrated the development of PLA-based drug delivery scaffolds for localized IBU release using AJS and ES. Both methods produced biocompatible, nanofibrillar structures with effective drug incorporation, controlled release, and comparable physicochemical properties. While ES offered greater stability, as evidenced by the release experiments, AJS provided a faster and more cost-effective alternative.

## Data Availability

Supporting data can be available upon direct request to the authors.

## References

[1] Chou P.Y., Lee D., Weng C.C., Wu R.C., Liao C.T., Liu S.J. (2022). Bone morphogenetic protein-, antimicrobial agent-, and analgesic-incorporated nanofibrous scaffolds for the therapy of alveolar clefts. Pharmaceutics.

[2] Wang F., Cai X., Shen Y., Meng L. (2023). Cell-scaffold interactions in tissue engineering for oral and craniofacial reconstruction. Bioact. Mater..

[3] Vazquez F.C., Chavarria D., Ortiz M., Guarino V., Alvarez M.A. (2022). 3D-Printed Tubular Scaffolds Decorated with Air-Jet-Spun Fibers for Bone Tissue Applications. Bioengineering.

[4] Wu D.T., Munguia J.G., Cho Y.W., Ma X., Song V., Zhu Z., Tran S.D. (2021). Polymeric scaffolds for dental, oral, and craniofacial regenerative medicine. Molecules.

[5] Cantón I., Mckean R., Charnley M., Blackwood K.A., Fiorica C., Ryan A.J., MacNeil S. (2010). Development of an Ibuprofen-releasing biodegradable PLA/PGA electrospun scaffold for tissue regeneration. Biotechnol. Bioeng..

[6] Collins M.N., Ren G., Young K., Pina S., Reis R.L., Oliveira J.M. (2021). Scaffold fabrication technologies and structure/function properties in bone tissue engineering. Adv. Funct. Mater..

[7] Hasnain M.S., Ahmad S.A., Chaudhary N., Hoda M.N., Nayak A.K., Inamuddin A.M., Mohammad A. (2019). Biodegradable polymer matrix nanocomposites for bone tissue engineering. Applications of Nanocomposite Materials in Orthopedics.

[8] Zhang J., Wehrle E., Rubert M., Muller R. (2021). 3D bioprinting of human tissues: Biofabrication, bioinks, and bioreactors. Int. J. Mol. Sci..

[9] Zhao Y., Liu Y., Kang S., Sun D., Liu Y., Wang X., Lu L. (2024). Peripheral nerve injury repair by electrical stimulation combined with graphene-based scaffolds. Front. Bioeng. Biotechnol..

[10] Gao Y., Duan J., Dang X., Yuan Y., Wang Y., He X., Bai R., Ye X.Y., Xie T. (2023). Design, synthesis and biological evaluation of novel histone deacetylase (HDAC) inhibitors derived from β-elemene scaffold. J. Enzyme Inhib. Med. Chem..

[11] Che D., Feng Y., Wei C., Zhou X., Zhang J., Shi Y., Wang L. (2021). Research in tissue engineering scaffold materials for alveolar bone repair. Crit. Rev. Biomed. Eng..

[12] Turk S., Altinsoy I., Celebi G., Ipek M., Ozacar M., Bindal C. (2018). 3D porous collagen/functionalized multiwalled carbon nanotube/chitosan/hydroxyapatite composite scaffolds for bone tissue engineering. Mater. Sci. Eng. C Mater. Biol. Appl..

[13] Batool F., Morand D.N., Thomas L., Bugueno I., Aragon J., Irusta S., Keller L., Benkirane-Jessel N., Tenenbaum H., Huck O. (2018). Synthesis of a novel electrospun polycaprolactone scaffold functionalized with ibuprofen for periodontal regeneration: An in vitro and In vivo study. Materials.

[14] Albanés-Ojeda E.A., Calderón-Olvera R.M., García-Hipólito M., Chavarría-Bolaños D., Vega-Baudrit R., Álvarez-Pérez M.A., Alvarez-Fregoso O. (2020). Physical and chemical characterization of PLA nanofibres and PLA/ZrO_2_ mesoporous composites synthesized by air-jet spinning. Indian J. Fibre Text. Res..

[15] Bharadwaz A., Jayasuriya A.C. (2020). Recent trends in the application of widely used natural and synthetic polymer nanocomposites in bone tissue regeneration. Mater. Sci. Eng. C Mater. Biol. Appl..

[16] Vazquez-Vazquez F.C., Chanes-Cuevas O.A., Masuoka D., Alatorre J.A., Chavarria-Bolaños D., Vega-Baudrit J.R., Serrano-Bello J., Álvarez-Pérez M.A. (2019). Biocompatibility of developing 3D-printed tubular scaffold coated with nanofibers for bone applications. J. Nanomater..

[17] Singhvi M.S., Zinjarde S.S., Gokhale D.V. (2019). Polylactic acid: Synthesis and biomedical applications. J. Appl. Microbiol..

[18] Safavi A.S., Karbasi S. (2024). A new path in bone tissue engineering: Polymer-based 3D-printed magnetic scaffolds (a comprehensive review of in vitro and in vivo studies). J. Biomater. Sci. Polym..

[19] Polak M., Karbowniczek J.E., Stachewicz U. (2024). Strategies in Electrospun Polymer and Hybrid Scaffolds for Enhanced Cell Integration and Vascularization for Bone Tissue Engineering and Organoids. Rev. Nanomed. Nanobiotechnol..

[20] Venkata Prathyusha E., Gomte S.S., Ahmed H., Prabakaran A., Agrawal M., Chella N., Alexander A. (2025). Nanostructured polymer composites for bone and tissue regeneration. Int. J. Biol. Macromol..

[21] Buj-Corral I., Sanz-Fraile H., Ulldemolins A., Tejo-Otero A., Domínguez-Fernández A., Almendros I., Otero J. (2022). Characterization of 3D printed metal-PLA composite scaffolds for biomedical applications. Polymers.

[22] Carriles J., Nguewa P., González-Gaitano G. (2023). Advances in Biomedical applications of Solution Blow Spinning. Int. J. Mol. Sci..

[23] Atif R., Khaliq J., Combrinck M., Hassanin A.H., Shehata N., Elnabawy E., Shyha I. (2020). Solution blow spinning of polyvinylidene fluoride based fibers for energy harvesting applications: A review. Polymers.

[24] Kramar A., Luxbacher T., González-Benito J. (2023). Solution blow co-spinning of cellulose acetate with poly(ethylene oxide). Structure, morphology, and properties of nanofibers. Carbohydr. Polym..

[25] Molfino H., Gonzales M., Alcalde-Yañez A., Valverde-Morón V., Villanueva-Salvatierra D. (2020). Electrospinning: Advances and applications in the field of biomedicine. Rev. Fac. Med. Hum..

[26] Madruga L.Y.C., Kipper M.J. (2022). Expanding the Repertoire of Electrospinning: New and Emerging Biopolymers, Techniques, and Applications. Adv. Healthc. Mater..

[27] Sabzekar M., Pourafshari M., Khayet M., García-Payo C., Mortazavi S.M., Golmohammadi M. (2023). Development of Novel Electrospun Fibers Based on Cyclic Olefin Polymer. Nanomaterials.

[28] Korat P.S., Kapupara P.P. (2017). Local infiltration of the surgical wound with levobupivacaine, ibuprofen, and epinephrine in postoperative pain: An experimental study. Biomed. Pharmacother..

[29] Hamed R., AbuRezeq A., Tarawneh O. (2018). Development of Hydrogels, Oleogels and Bigels as Local Drug Delivery Systems for Periodontitis. Drug Dev. Ind. Pharm..

[30] Mohiti-Asli M., Saha S., Murphy S.V., Gracz H., Pourdeyhimi B., Atala A., Loboa E.G. (2017). Ibuprofen loaded PLA nanofibrous scaffolds increase proliferation of human skin cells in vitro and promote healing of full thickness incision wounds in vivo. J. Biomed. Mater. Res. B Appl. Biomater..

[31] Romsing J., Moiniche S., Ostergaard D., Dahl J.B. (2000). Local infiltration with NSAIDs for postoperative analgesia: Evidence for a peripheral analgesic action. Acta Anaesthesiol. Scand..

[32] Sarma H., Joshi S.J., Prasad R., Jampilek J. (2021). Biobased Nanotechnology for Green Applications.

[33] Kumar P., Dehiya B.S., Sindhu A. (2019). Ibuprofen-Loaded CTS/nHA/nBG Scaffolds for the Applications of Hard Tissue Engineering. Iran Biomed. J..

[34] Hersh E.V., Moore P.A., Grosser T., Polomano R.C., Farrar J.T., Saraghi M., Juska S.A., Mitchell C.H., Theken K.N. (2020). Nonsteroidal anti-inflammatory drugs and opioids in postsurgical dental pain. J. Dent. Res..

[35] Khan H., Sharma K., Kumar A., Kaur A., Singh T.G. (2022). Therapeutic implications of cyclooxygenase (COX) inhibitors in ischemic injury. Inflamm. Res..

[36] Keb C.A.F. (2022). Mechanism of NSAIDs and derived drugs for pain and inflammation control. Use of anti-inflammatories in odontology. Rev. ADM.

[37] Radi Z.A., Khan K.N. (2019). Cardio-renal safety of non-steroidal anti-inflammatory drugs. J. Toxicol. Sci..

[38] Bindu S., Mazumder S., Bandyopadhyay U. (2020). Non-steroidal anti-inflammatory drugs (NSAIDs) and organ damage: A current perspective. Biochem. Pharmacol..

[39] Rainsford K.D. (2009). Ibuprofen: Pharmacology, efficacy and safety. Inflammopharmacology.

[40] Grzybowska K., Grzybowski A., Knapik-Kowalczuk J., Chmiel K., Woyna-Orlewicz K., Szafraniec-Szczƒôsny J., Antosik-Rogóż A., Jachowicz R., Kowalska-Szojda K., Lodowski P. (2020). Molecular dynamics and physical stability of ibuprofen in binary mixtures with an acetylated derivative of maltose. Mol. Pharm..

[41] Jasim H.H. (2015). Determination of Ibuprofen in Aqueaus Solutions and Pharmacetical Preparations by UV-VIS Spectrophotometric. Al-Nahrain J. Sci..

[42] Blanca-López N., Soriano V., Garcia E.M., Canto G., Blanca M. (2019). NSAID-induced reactions: Classification, prevalence, impact, and management strategies. J. Asthma Allergy.

[43] Lee D.J., Lee S., Kim I.W. (2012). Effects of humidity and surfaces on the melt crystallization of ibuprofen. Int. J. Mol. Sci..

[44] Koperwas K., Tu W., Affouard F., Adrjanowicz K., Kaskosz F., Paluch M. (2021). Pressure dependence of the crystallization rate for the S-enantiomer and a racemic mixture of ibuprofen. Cryst. Growth Des..

[45] Bolla P.K., Clark B.A., Juluri A., Cheruvu H.S., Renukuntla J. (2020). Evaluation of formulation parameters on permeation of ibuprofen from topical formulations using Strat-M-membrane. Pharmaceutics.

[46] Uysal İ., Eratilla V., Topbaş C., Ergül İ., Çelik Y. (2022). Comparison of local and systemic ibuprofen for relief of postoperative pain in symptomatic teeth with apical periodontitis. Med. Sci. Monit..

[47] Wade A.G., Crawford G.M., Young D., Corson S., Brown C. (2019). Comparison of diclofenac gel, ibuprofen gel, and ibuprofen gel with levomenthol for the topical treatment of pain associated with musculoskeletal injuries. J. Int. Med. Res..

[48] Sun C., Zou L., Xu Y., Wang Y. (2020). Ibuprofen-loaded poly(lactic acid) electrospun mats: The morphology, physicochemical performance, and in vitro drug release behavior. Macromol. Mater. Eng..

[49] Serrano-Garcia W., Bonadies I., Thomas S.W., Guarino V. (2023). New Insights to Design Electrospun Fibers with Tunable Electrical Conductive-Semiconductive Properties. Sensors.

[50] Salama A., Tolba E., Saleh A.K., Cruz-Maya I., Alvarez-Perez M.A., Guarino V. (2024). Biomineralization of Polyelectrolyte-Functionalized Electrospun Fibers: Optimization and In Vitro Validation for Bone Applications. Biomimetics.

[51] Chavarría D., Vega J., Cerda B., Pozos A., Montero M., Guarino V., Ålvarez M. (2021). Translation of Tissue Engineering Aproach from Clinics. Current Advances in Oral and Craniofacial Tissue Engineering.

[52] Belmessaoud N.B., Bouslah N., Haddadine N. (2020). Clay/(PEG-CMC) biocomposites as a novel delivery system for ibuprofen. J. Polym. Sci. Eng..

[53] Livecchi L., McAuley W.J., Kerai-Varsani L. (2021). The use of optical differential scanning calorimetry to investigate ibuprofen miscibility in polymeric films for topical drug delivery. Eur. J. Pharm. Biopharm..

[54] Ha M.W., Paek S.M. (2021). Recent Advances in the Synthesis of Ibuprofen and Naproxen. Molecules.

[55] Li Q., Choong C. (2013). Three-Dimensional Scaffolds for Tissue Engineering Applications: Role of Porosity and Pore Size. Tissue Eng. Part B Rev..

[56] Solarz D., Witko T., Karcz R., Malagurski I., Ponjavic M., Levic S., Nesic A., Guzik M., Savicg S., Nikodinovic-Runic J. (2023). Biological and physiochemical studies of electrospun polylactid/polyhydroxyoctanoate PLA/P(3HO) scaffolds for tissue engineering applications. RSC Adv..

[57] Granados-Hernández M.V., Serrano-Bello J., Montesinos J.J., Alvarez-Gayosso C., Medina-Velázquez L.A., Alvarez Fregoso O., Alvarez-Perez M.A. (2018). In vitro and in vivo biological characterization of poly(lactic acid) fiber scaffolds synthesized by air jet spinning. J. Biomed. Mater. Res. B Appl. Biomater..

[58] Laffite F.M. (2023). Diseño de Membranas de Ácido Poliláctico para Liberación Controlada de Tramadol.

[59] Suarez-Franco J.L., Vázquez-Vázquez F.C., Pozos-Guillen A., Montesinos J.J., Alvarez-Fregoso O., Alvarez-Perez M.A. (2018). Influence of diameter of fiber membrane scaffolds on the biocompatibility of hPDL mesenchymal stromal cells. Dent. Mater. J..

[60] González I. (2022). Cristalización, Caracterización Estructural y Estudio de las Interacciones del Cocristal Ibuprofeno-Nicotinamida.

[61] Gómez-Pachón E., Graziano V., Montiel R., Cardenas-Aguazaco W., Ochica A. (2019). Effects of heat treatment and the method of collection on the crystal structure of the poly lactic acid electrospinned nanofibers. Rev. Chil. Ing..

[62] Posada E. (2014). Validación de la Metodología de Contenido Químico de Ibuprofeno en Tabletas por Calorimetría Diferencial de Barrido.

[63] Jiménez-Minota J. (2017). Evaluación de la Cinética de Liberación de un Fármaco Modelo con Clasificación Biofarmacéutica Clase ii, Desde Matrices Comprimidas Compuestas por Materiales Poliméricos Aniónicos.

[64] Bonillo-Martinez A. (2017). Desarrollo de Comprimidos de Liberaci√≥n Controlada con una Poliesteramida Derivada de L-Alanina, PADAS.

[65] Rabelo L.H., Munhoz R.A., Marini J., Maestrelli S.C. (2022). Development and Characterization of PLA Composites with High Contents of a Brazilian Refractory Clay and Improved Fire Performance. Mater. Res..

[66] Namazi Z., Jafarzadeh-Kashi T.S., Erfan M., Najafi F., Bakhtiari L., Ghodsi S.R., Farhadnejad H. (2019). Synthesis and Characterization of Ibuprofen-mesoporous Hydroxyapatite Nanohybrid as a Sustained Drug Delivery System. Iran J. Pharm. Res..

[67] Khaskheli A.R., Sirajuddin S.T., Mahesar S.A., Kandhro A.A., Kalwar N.H., Mallah M.A. (2013). Estimation of ibuprofen in urine and tablet formulations by transmission Fourier Transform Infrared spectroscopy by partial least square. Spectrochim Acta A Mol. Biomol. Spectrosc..

[68] Ramukutty S., Ramachandran E. (2011). Growth, spectral and thermal studies of ibuprofen crystals. Cryst. Res. Technol..

[69] Shoaib Q., Abbas N., Irfan M., Hussain A., Sohail M., Hussain S., Latif S., Bukhari N. (2018). Development and Evaluation of Scaffold Based Nanosponge Formulation for Controlled Drug Delivery of Naproxen and Ibuprofen. Trop. J. Pharm. Res..

[70] Lemraski E.G., Alibeigi S., Abbasi Z. (2022). Ibuprofen @silver loaded on poly(vinyl alcohol)/chitosan co-polymer scaffold as a novel drug delivery system. Mater. Today Commun..

[71] Rodríguez M. (2018). Preparación de Biocatalizadores Basados en Lipasa Inmovilizada. Aplicación en la Resolución de Ibuprofeno Racémico.

[72] Perneger T.V. (2021). How to use likelihood ratios to interpret evidence from randomized trials. J. Clin. Epidemiol..

[73] Elston D.M. (2022). Likelihood ratios. J. Am. Acad. Dermatol..

[74] Riggin C.N., Qu F., Kim D.H., Huegel J., Steinberg D.R., Kuntz A.F., Bernstein J. (2017). Electrospun PLGA Nanofiber Scaffolds Release Ibuprofen Faster and Degrade Slower After In Vivo Implantation. Ann. Biomed. Eng..

[75] Lima A.F., Pegorin G.S., Miranda M.C.R., Cachaneski-Lopes J.P., Silva W.M., Borges F.A., Guerra N.B., Herculano R.D., Batagin-Neto A. (2021). Ibuprofen-loaded biocompatible latex membrane for drug release: Characterization and molecular modeling. J. Appl. Biomater. Funct. Mater..

[76] Melguizo-Rodríguez L., Costela-Ruiz V.J., Manzano-Moreno F.J., Illescas-Montes R., Ramos-Torrecillas J., García-Martínez O., Ruiz C. (2018). Repercussion of nonsteroidal anti-inflammatory drugs on the gene expression of human osteoblasts. PeerJ.

[77] Al-Waeli H., Reboucas A.P., Mansour A., Morris M., Tamimi F., Nicolás B. (2021). Non-steroidal anti-inflammatory drugs and bone healing in animal models-a systematic review and meta-analysis. Syst. Rev..

[78] García-Martínez O., De Luna-Bertos E., Ramos-Torrecillas J., Manzano-Moreno F.J., Ruiz C. (2015). Repercussions of NSAIDS drugs on bone tissue: The osteoblast. Life Sci..

[79] Limami Y., Leger D.Y., Liagre B., Pécout N., Viana M. (2021). Ibuprofen-loaded calcium phosphate granules: A new bone substitute for local relieving symptoms of osteoarthritis. Eur. J. Pharm. Sci..

[80] Pergolizzi J.V., Magnusson P., LeQuang J., Gharibo C., Varrassi G. (2020). The pharmacological management of dental pain. Expert Opin. Pharmacother..

[81] García-Ramírez P.E., González-Rodríguez S.G., Soto-Acevedo F., Brito-Zurita O.R., Cabello-Molina R., López-Morales C.M. (2018). Dolor postoperatorio: Frecuencia y caracterización del manejo. Rev. Colomb. Anestesiol..

[82] Abella-Palacios P., Arias-Amézquita F., Barsella A.R., Hernández-Porras B.C., Narazaki D., Salomón-Molina P.A. (2021). Inadequate management of acute postoperative pain: Prevalence, prevention, and consequences. Review of the situation in Latin America. Rev. Mex. Anestesiol..

[83] Al-Bayati O., Font K., Soldatos N., Carlson E., Parsons J., Powell C.A. (2021). Evaluation of the need to prescribe opioid medication to control postsurgical pain of different periodontal/oral surgeries. J. Periodontol..

[84] Forget P. (2019). Opioid-free anaesthesia. Why and how? A contextual analysis. Anaesth Crit. Care Pain Med..

[85] Malamed S.F. (2023). Manejo del dolor después de un traumatismo dental y procedimientos quirúrgicos. Dent. Traumatol..

